# Amino acid sequence alignment of vertebrate CAPN3/calpain-3/p94

**DOI:** 10.1016/j.dib.2015.09.021

**Published:** 2015-10-04

**Authors:** Yasuko Ono, Hiroyuki Sorimachi

**Affiliations:** Calpain Project, Department of Advanced Science for Biomolecules, Tokyo Metropolitan Institute of Medical Science (IGAKUKEN), 2-1-6 Kamikitazawa, Setagaya-ku, Tokyo 156-8506, Japan

## Abstract

CAPN3 is a calpain superfamily member that is predominantly expressed in skeletal muscle. So far, clear CAPN3 orthologs were found only in vertebrates. CAPN3 is a unique protease in that it undergoes extremely rapid and exhaustive autolysis and that autolyzed fragments spontaneously associate each other to reconstitute the proteolytic activity. These unique properties of CAPN3 are dependent on IS1 and IS2, two CAPN3-characterizing sequences that do not exist in other calpains or any other proteases. To understand how IS1 and IS2 are conserved among vertebrates, this data article provides amino acid sequence alignment of representative vertebrate CAPN3s. For further analysis and discussion, see Ono et al. [1]

**Specifications table**

**Table:** 

Subject area	*Biology*
More specific subject area	*Molecular evolution*
Type of data	*Figure*
How data was acquired	*Retrieved from public databases*
Data format	*Analyzed*
Experimental factors	*Each amino acid sequences were retrieved from NCBI and/or Ensemble database shown below.*
Experimental features	*Sequences were aligned using MOE Ver.2014.09 and GENETYX Ver.12*
Data source location	*NCBI:*http://www.ncbi.nlm.nih.gov/
*Ensemble:*http://asia.ensembl.org/
Data accessibility	*Data with article*

**Value of the data**

• CAPN3 is a unique protease and its molecular evolution is intriguing to understand its unique features.
• Sequence alignment is essential to study molecular evolution.
• CAPN3 has two characteristic regions, IS1 and IS2, which are involved in unique features of CAPN3
• Sequence alignment defines these regions, and their conservation.

## 1 Data

Representative vertebrate CAPN3 sequences were aligned using MOE Ver. 2014.09 and GENETYX Ver. 12 software. Based on the alignment in this data, Figs. 5, 6, and 7 and Table 3 of Ref. [1] were made.

## 2 Experimental design, materials and methods

Sequence data were retrieved from NCBI (http://www.ncbi.nlm.nih.gov/) and Ensemble (http://asia.ensembl.org/) databases. Representative vertebrates that have complete or close to complete amino acid sequence(s) for CAPN3 were selected for alignment. These sequences were aligned by Protein Align function of MOE Ver.2014.09 software using default parameters, and then refined using Parallel Editor of GENETYX Ver.12.

## Figures and Tables

**Fig. S1:**
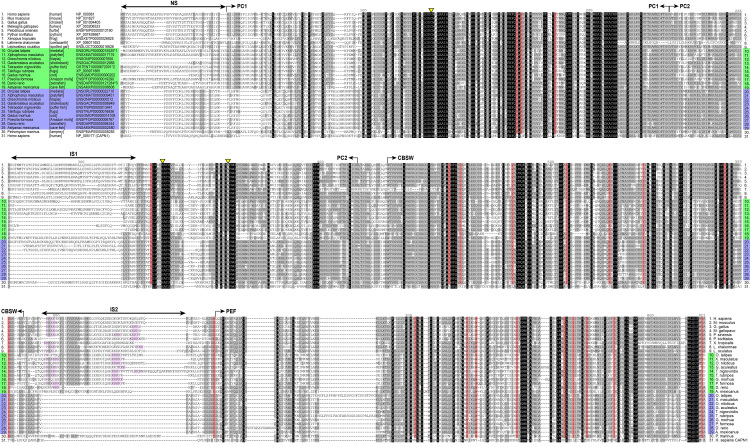
Representative vertebrate CAPN3 sequences, which are indicated in Fig. 5 of our *Biochimie* article, were aligned using MOE Ver. 2014.09 and GENETYX Ver. 12 software. Residue numbers of human CAPN3 are given above the sequences. Residues conserved in all the sequences, all except human CAPN1, and in more than half the sequences are indicated by black reversed fonts, red, and gray shadow, respectively. Teleosts have two CAPN3 genes, *Capn3a* and *Capn3b*, which are indicated by pale blue and green, respectively. Yellow triangles: active site residues. Pink shadow in IS2: three or more than three successive K or R residues. *1 Sequence was revised using XP_004083493; no exon sequence has been found that correspond to exon 15 in humans. *2 Sequence was revised using EST sequences. *3 No exon sequence has been found that correspond to exon 16 in humans.
